# Communication among health professionals using newborn technology for care: an exploratory scoping review

**DOI:** 10.1136/bmjoq-2025-003501

**Published:** 2025-09-16

**Authors:** Gloria Karungo Ngaiza, Dorothy Oluoch, Catherine Molyneux, Catherine Pope, Caroline Jones

**Affiliations:** 1 Health Systems Collaborative, Nuffield Department of Medicine, University of Oxford, Oxford, UK; 2 Health Systems and Research Ethics, KEMRI-Wellcome Trust Research Programme, Nairobi, Kenya; 3 Nuffield Department of Primary Healthcare Sciences, University of Oxford, Oxford, UK

**Keywords:** Child, Equipment and Supplies, Communication, Healthcare quality improvement

## Abstract

Neonatal technologies can significantly improve care quality and reduce newborn deaths. However, their successful implementation in complex health system contexts requires effective communication among health professionals. A comprehensive understanding of communication among professionals using newborn technologies is needed to inform technology implementation. We conducted a scoping review of the current literature. We searched the grey literature and online resources, including PubMed, Web of Science, Scopus, Embase, Cochrane Library and Google Scholar, for articles. We included English literature that discussed the use of technology in newborn care. 13 papers met the inclusion criteria. We analysed the findings using a thematic approach. 11 of the 13 papers included were based on research conducted in low-income and middle-income countries (LMICs), with continuous positive airway pressure being the most frequently covered technology. The communication information was limited, as these topics were just one of many themes in the papers. Most studies focused on nurses, encompassing aspects of communication such as knowledge sharing and interactions during patient management, monitoring and documentation. However, there was little detail on the nature of these interactions or where they occurred. Factors influencing communication included physical contexts such as infrastructure, socio-organisational contexts such as hierarchy and levels of skills, and technology-related factors such as perceived usefulness and ease of use. If and how these factors interacted with each other to shape technology-related communication was unclear. We highlight gaps in the literature on communication among health professionals using newborn technology for care. We stress the importance of carefully examining the physical and socio-organisational contextual factors and technology-specific attributes that shape communication in all settings, including LMICs. Research aiming to better understand the context of technology implementation will support the successful implementation of potentially life-saving technologies.

**WHAT IS ALREADY KNOWN ON THIS TOPIC**

**WHAT THIS STUDY ADDS**

- An overview of communication among health professionals using newborn technologies, covering what is communicated, the settings, the people involved and the factors influencing communication.

**HOW THIS STUDY MIGHT AFFECT RESEARCH, PRACTICE OR POLICY**

- This study identifies gaps in evidence on how the context of newborn technology implementation affects communication among health professionals, emphasising the need for further exploration of these factors.
- Understanding these contextual factors is essential for the successful adoption and implementation of newborn technologies, ensuring that communication dynamics are integrated into policy, facility design and training programmes.

## Introduction

 Neonatal mortality is a global health challenge, with 2.3 million neonatal deaths reported in 2022 alone despite a significant reduction from 5.0 million in 1990.^1^ Sub-Saharan Africa has the highest neonatal mortality rate at 27 deaths per 1000 live births. A baby born in sub-Saharan Africa is at 11 times greater risk of dying than one born in the lowest-mortality region.^1^

Affordable newborn technologies such as a phototherapy machine and a continuous positive airway pressure (CPAP) can significantly improve the quality of care and reduce preventable newborn deaths.^2 3^ These technologies are introduced in a complex health system^4^ whose context contributes to their integration.^5^ It is common for the implementation of these technologies in health facilities in low-income and middle-income countries (LMICs) to fail as a result of inadequate attention to important contextual factors such as staffing numbers and training. This leads to what is often referred to as ‘equipment graveyards’.^6^ Equipment graveyard refers to a collection of expensive technologies that remain unused due to high staff workloads, maintenance challenges, poor infrastructure and limited resources.^7 8^

Effective communication is essential in delivering newborn interventions in complex health systems. It is highlighted as one of the eight standards in the *2020* WHO Standards for Improving the Quality of Care for Small and Sick Newborns in Health Facilities.^9^ The WHO standards emphasise the pivotal function of effective communication in fostering collaboration among health professionals, facilitating regular and accurate information exchange, and averting miscommunication among professionals and families.^9^

There is a lack of a comprehensive understanding of communication among health professionals engaging with newborn technologies to inform their successful implementation. In this review, we focus on understanding technology-associated communication among health professionals in the newborn unit context.

## Methods

### Search strategy

We developed a protocol (online supplemental material 1) to guide the scoping review. The Joanna Briggs Institute Manual for Evidence Synthesis^10^ and Preferred Reporting Items for Systematic Review and Meta-Analysis extension for Scoping Reviews (PRISMA-ScR)^11^ (online supplemental material 2) informed the data collection and synthesis process.

The review answered these questions:

1. How do health professionals communicate while using technologies in newborn care settings? This includes examining who is involved, the content of the communication and the locations where these interactions occur.
2. What factors shape communication among health professionals using technologies in newborn care within hospital settings, and how do they impact this communication?

### Search strategy and source of literature

We conducted the first round of literature searches between June and August 2021 and updated the list in March 2025 to add any new papers published since August 2021. We searched relevant websites and seven databases: PubMed, Web of Science, Scopus, Embase, ERIC, Cochrane Library and Google Scholar.

We identified five keywords to guide the search for papers and the inclusion and exclusion criteria. These were communication, technology, health professionals, neonates and health facilities. The search terms used to identify articles on PubMed were:

(“communication” OR “interaction*”) AND (“Health professional*” OR “Health worker*” OR “workforce*” OR “human resource for health” OR “Healthcare worker*” OR “nurse*”) AND (“Technolog*” OR “innovation*” OR “medical?device*”) AND (“Neonat*” OR “new?born*” OR “bab?*” OR “below 28 days” OR “less than 28 days”) AND (“Hospital*” OR “ward*” OR “health cent?*” OR “neonatal unit” OR “new?born unit” OR facilit*”)

### Eligibility criteria

For this scoping review, we adopted the definition of communication used by the WHO, which refers to providing information, guidance and advice to protect the health of individuals.^12^ We included literature on communication among health professionals that considers any information exchange related to technology use. This includes, but is not limited to, training and learning to operate technology, documentation related to technology use, interactions during clinical decisions and patient monitoring, and other interactions connected to the use of newborn technology. We defined technology as an innovation, a device or a process introduced to change the existing practice or improve newborn care.^13^,^15^ We excluded papers on information and communication technology because we focused on the communication process related to technology use, not how technology functions as a communication medium.

As shown in table 1, we included the literature about all disciplines of health professionals working in neonatal care. We adopted the neonate definition of a baby from the day of delivery up to 28 days of life.^16^ We included literature on health facilities at all levels, including primary healthcare centres, district or first referral, and specialised and general hospitals as defined by the WHO.^17^

**Table 1:** The criteria for including studies in the scoping review

Inclusion criteria	
Period	No time limit on the research data collection or article publication.
Document type and location	Unpublished and published literature including empirical studies. Articles that discuss the use of one or multiple technologies in newborn care regardless of the geographical location.
Language	Articles written in English.
Research method	Studies that used qualitative, quantitative and mixed methods.

### Data charting and synthesis

We developed a data extraction management tool on Microsoft Excel, which captured information on included studies, including the author(s), year of publication, geographical location, methodology, technology type and limitations. Informed by previous studies, columns in the spreadsheet also documented findings based on identified essential areas in healthcare communication: groups of health professionals involved in the communication,^18^ ways of communicating,^18^,^20^ information content,^19 21^ time the communication took place and the quality^20^ and factors affecting communication.^18^,^20^

The first reviewer, GKN, screened the article titles to remove duplicates and used abstract review to determine the eligibility of articles. She read the full texts of articles that met the inclusion criteria and the references of key articles to scan for other eligible articles. Throughout the review process, the wider research team reviewed inclusion and exclusion criteria and discussed extracted data for quality assurance. Drawing on Braun and Clarke,^22^ we familiarised ourselves with data, generated codes and developed themes that were reviewed and refined.

## Results

**Figure 1:**
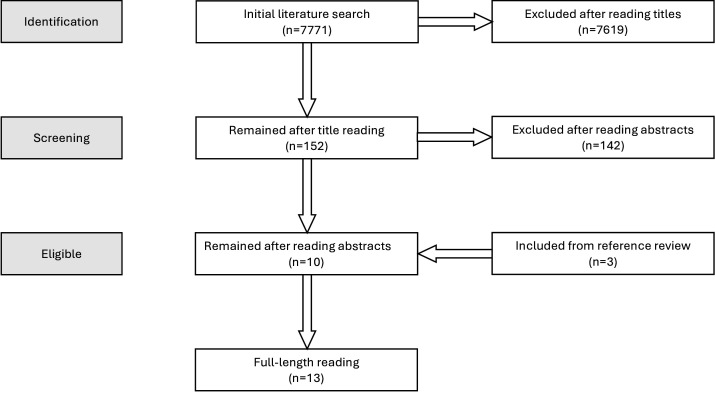
Scoping review chart highlighting the screening and selection process.

The first search yielded 7771 articles. After reading the titles, we excluded 7619 papers, including duplicates. We had 152 articles for abstract review. A total of 10 articles that met the review criteria passed to the final stage of full-length review. We also included three articles from the relevant article references to make the final list of papers 13. These 13 papers reported 12 studies with publication years between 2015 and 2024. Figure 1 summarises the process of choosing scoping review articles.

Characteristics of articles included in the review are in table 2.

**Table 2:** Articles included in the scoping review

Author, year	Title	Country	Technology	Methods
Dewez *et al*.^28^ 2018	Healthcare workers’ views on the use of continuous positive airway pressure (CPAP) in neonates: a qualitative study in Andhra Pradesh, India.	India	CPAP	18 in-depth interviews with doctors. 8 focus group discussions with 51 nurses. 15 neonatal units in district and college hospitals.
Anton *et al*.^30^ 2019	Functionality and acceptability of a novel non-invasive neonatal heart rate monitoring device: a qualitative study of healthcare professionals.	United Kingdom	Non-invasive neonatal heart rate monitoring device	Semistructured interview with 21 paediatricians, midwives and neonatal nurses. 3 newborn intensive care units and a labour ward.
Kinshella *et al*.^24^ 2020	Scaling up newborn care technologies from tertiary-level to secondary-level hospitals in Malawi: an implementation case study of health professional perspectives on bubble CPAP.	Malawi	CPAP	A secondary analysis of interviews with 46 health professionals (nurses, clinicians, district health management). 1 tertiary and 3 secondary-level hospitals.
Nabwera *et al*.^31^ 2020	Sometimes you are forced to play God horizontal ellipsis: a qualitative study of healthcare worker experiences of using continuous positive airway pressure in newborn care in Kenya.	Kenya	CPAP	16 key informant interviews and 15 focus group discussions among 100 healthcare workers in Kenya. 19 secondary and tertiary hospitals (15 public, 2 private and 2 mission).
Nyondo-Mipando *et al*.^23^ 2020	Barriers and enablers of implementing bubble continuous positive airway pressure (CPAP): perspectives of health professionals in Malawi.	Malawi	CPAP	46 in-depth interviews with nurses, clinicians and district health managers. 3 district hospitals and 1 tertiary hospital.
Aneji *et al*.^32^ 2020	Implementing bubble continuous positive airway pressure in a lower-middle-income country: a Nigerian experience.	Nigeria	CPAP	In-depth interviews and focus group discussions with healthcare workers and hospital administrators (unspecified number). 7 established hospitals.
Asibon *et al*.^33^ 2020	Using a peer mentorship approach improved the use of neonatal continuous positive airway pressure and related outcomes in Malawi.	Malawi	CPAP	Assessment and self-evaluation checklist of 113 nurses. 10 secondary and one tertiary hospital.
Lu and Li^34^ 2020	Action research on neonatal nursing staff: experiences caring for bubble continuous positive airway pressure patients.	Taiwan	CPAP	Interviews with 20 special care paediatric nurses. 1 children’s hospital.
Kinshella, M. W., *et al*^26^ 2022	Qualitative study exploring the feasibility, usability and acceptability of neonatal continuous monitoring technologies at a public tertiary hospital in Nairobi, Kenya.	Kenya	3 continuous monitoring technologies (Monitors respiration rate, heart rate, pulse, CO2 levels and body movement)	In-depth interviews with 27 participants (6 nurses, 2 study nurses, 2 clinical officers, 2 physicians, 5 healthcare administrators and 10 caregivers). 1 tertiary hospital
Kinshella *et al*.^35^ 2022	Challenges and recommendations to improve implementation of phototherapy among neonates in Malawian hospitals.	Malawi	Phototherapy	Mixed-method study comprised of a facility assessment and 31 qualitative interviews with nurses, clinicians, paediatric physicians and district health managers and caregivers. 3 secondary-level hospitals.
Agarwal *et al*.^29^ 2023	Feasibility and acceptability of the paediatric pulse oximeter in integrated management of neonatal and childhood illnesses (IMNCI) services by public health facilities: a qualitative study in rural Western India.	India	Pulse oximeter	In-depth interview with Medical Officers (n=6) and Auxiliary nurse midwives (n=6). 6 primary healthcare centres.
Mdoe *et al*.^25^ 2023	Healthcare workers’ perceptions on the ‘SaferBirths Bundle of Care’: a qualitative study.	Tanzania	SaferBirths Bundle of Care Moyo (Fetal Heart rate), NeoBeat (Fetal heart rate metre) and upright bag mask	21 focus group discussions with 106 midwives and doctors and 43 individual interviews with midwives, doctors and staff in leadership roles. 4 regional hospitals, 15 district hospitals and 2 health centres.
Nyondo-Mipando, A. L., *et al.*^27^ 2024	Factors influencing the implementation of infant warming devices among healthcare workers in Malawian hospitals.	Malawi	Infant warming devices including radiant warmers, hot-cots, heaters, portable warmers and incubators	19 interviews with nurses, clinicians, paediatricians, district health management members. 1 tertiary and 1 secondary level hospitals.

Of the 13 articles in the scoping review, there were 12 studies, with one having two papers.^23 24^ 10 studies were from LMICs, while two were from high-income countries. As shown in table 2, these studies reported on seven different technologies: CPAP in six studies, reported in seven papers and one study each reported on phototherapy, pulse oximeter, SaferBirths Bundle of Care, non-invasive neonatal heart rate monitoring device, infant warming devices and continuous monitoring devices. Most studies examined a single technology, except for three studies that discussed a bundle of technologies.^25^,^27^ While one study focuses explicitly on CPAP, participants also mentioned a ventilator in their responses.^28^

While communication was not the primary focus in any of the studies, it arose as a key issue for the participants and the authors in investigating issues such as functionality, acceptability, feasibility and perceptions. For example, communication emerged as a challenge in a study to examine the feasibility and acceptability of the paediatric pulse oximeter in the integrated management of neonatal and childhood illnesses services across six primary health facilities in India.^29^ Similarly, a study by Anton *et al* examining the functionality and acceptability of a novel non-invasive neonatal heart rate monitoring device in three neonatal intensive care units (NICUs) and one labour ward in the UK reported on communication among health professionals.^30^

### Who is involved in communication?

Studies included in the review reported communication among various groups of health professionals in the newborn unit. While two studies did not specify the disciplines of health professionals investigated,^31 32^ 10 studies provided specific details. Four studies reported on health administrators or leaders as well as nurses and doctors.^2325^,^27^ Three studies reported on both nurses and doctors,^28^,^30^ two solely on nurses^33 34^ and one on communication involving health professionals and parents.^24^ Nurses and doctors were the health professionals most frequently studied (in 10 and 8 studies, respectively).

### What is the content of communication, and how was it communicated?

We categorised what health professionals communicated into two groups: (1) knowledge sharing and (2) patient management, monitoring and documentation.

### Knowledge sharing

Knowledge sharing about technology use forms a key part of communication during training. Various approaches to training on medical technologies were reported. For example, one approach was verbal, using trained health professionals to cascade knowledge transfer from the centre to peripheral facilities through a Training of Trainers. In Malawi, Kinshella *et al* drew on secondary hospital data to report that nurses from district hospitals received training on the use of CPAP at the tertiary hospital nursery and then cascaded this knowledge to their colleagues.^24^ Similarly, a qualitative study examining the utilisation of CPAP in newborn care across 19 hospitals in Kenya documented that senior staff members underwent comprehensive training sessions.^31^ Subsequently, they disseminated their knowledge and skills to other health professionals during in-house educational meetings. This study found that some health professionals missed out on the training, adversely impacting their decision-making regarding CPAP use. For example, there were instances where CPAP treatment was prematurely discontinued when patients were transferred to different staff members for care. Where training did not align with existing practices in the trainees’ facilities, there was disagreement among health professionals regarding eligibility for CPAP treatment.^24^

Written communication was used to help facilitate improvements in practice. Health professionals documented and used the information to plan simulation scenario sessions to enhance clinical skills.^25^ A study conducted in Nigeria investigated the implementation of CPAP in seven hospitals, where the training involved designated champions within each hospital facilitating hands-on interpersonal training sessions.^32^ There were efforts to establish an online group for virtual written communication via WhatsApp among the champions during the initial stages of CPAP implementation. However, these efforts were unsuccessful. This failure was attributed to the health professionals’ underutilisation of the platform across all sites.

Another way of sharing knowledge reported in the three studies^26 27 30^ was using guidelines. In the study conducted in NICUs and a labour ward in the UK, participants were reported as recommending printed visual aids such as instruction sheets on resuscitation or preattached instructions on the non-invasive neonatal heart rate monitoring device as a means of communication.^30^ In the Malawian research study undertaken in three secondary-level hospitals, written guidelines were available to assist health professionals in phototherapy. However, their use was reported to be inconsistent.^26 35^ Similarly, health professionals in Malawi reported unclear guidelines on discontinuing infant warming devices. This lack of direction led to wasted time in debates about the technology’s use rather than focusing on patient care.^27^

### Patient management, monitoring and documentation

Communication among health professionals included information exchange on patient management. Participation in decisions regarding technology use varied across health professionals involved in the review and the study context. In a study to assess the peer mentorship approach to improve the use of CPAP in 11 hospitals in Malawi, health professionals discussed and made decisions to initiate CPAP for eligible newborn babies.^33^ In four hospitals in Malawi, nurses in secondary-level hospitals contributed to the decision-making process regarding CPAP usage. In contrast, in the tertiary hospital, autonomy in decision-making was primarily accorded only to doctors.^24^

Health professionals also communicated concerning monitoring and documenting the progress of babies whose care involved using technologies. For example, in the Malawian study examining the implementation of CPAP in one tertiary and three secondary hospitals, it was found that nurses, clinicians and the district health management discussed the regular monitoring of breathing required for CPAP patients. This monitoring commenced within the first 15 min of CPAP initiation and was supposed to be followed by hourly checks and subsequently every 4–6 hours.^33^

### When did health professionals communicate?

Studies provided limited information concerning the timing of communication among health professionals regarding technology. However, a Malawian study on the implementation of CPAP in one tertiary and three secondary hospitals reported that interactions among health professionals were more challenging at night due to reduced staff and increased workload.^23 24^

### What factors shape communication quality?

#### Physical context

Infrastructure in newborn care influenced communication among health professionals involved with newborn technologies. For example, the availability of electricity to power the machines played a role in health professionals’ communication. In the Nigerian study, none of the seven hospitals had a consistent electricity supply. Before implementing CPAP, only one hospital had nearly continuous power availability. However, this hospital encountered significant disruptions when its NICU underwent renovations without proper coordination, leading to a 50% decrease in electricity availability and reliability. As a result, health professionals extended their communication to involve people responsible for managing the hospital’s power. The constant disruption also led to the decision not to use CPAP.^32^

Similarly, another study reported power outages in three secondary-level hospitals in Malawi. The challenge of the inconsistency of electricity supply was a significant issue that altered health professionals’ communication on decisions to use or not use phototherapy. Lack of power led to health professionals deciding not to start phototherapy in newborn care.^35^ In another study from Malawi, nurses and clinicians reported communicating on the decisions to use CPAP, which was affected by the daily electricity outages, often lasting for several hours. Even with a generator, there were delays and challenges due to fuel shortages.^24^ In a study in Kenya, a similar challenge with electricity led health professionals to spend the already limited time engaging with people outside the newborn unit to discuss what decisions to make based on the available power alternative from a generator.^31^

### Organisational context

The organisational context of newborn units, including the support provided to health professionals to enhance their expertise, played a role in health professionals’ communication around technology. For instance, in the Malawi study on CPAP implementation, the hierarchical culture contributed to clinicians holding greater authority in technology-associated discussions and decision-making than nurses. During critical situations, trained nurses could initiate CPAP, but they would subsequently consult a clinician for follow-up.^23 24^ By contrast, in the Tanzanian study on safe births, the authors documented how a friendly atmosphere that allowed health professionals to learn from mistakes without criticism encouraged them to use technology. The presence of champions among the health professionals who provided instruction on technology use was pivotal in encouraging health professionals to communicate and engage with the Safe Birth Bundle of Care technologies.^25^

Several studies reported an inadequate number of health professionals to shape communication and decisions about using technologies. For instance, health professionals in the Malawi study conducted in one tertiary and three secondary hospitals described how the low number of health professionals led to nurses being reassigned to labour and postnatal wards. The small number of health professionals contributed to the reluctance to make the decision to initiate CPAP treatment because of the high interactions and documentation needed during its use.^24^ Similarly, in the Kenyan study, health professional shortages and strikes contributed to discussions on decisions not to use CPAP. This was because nurses linked CPAP to the additional interactions and documentation needed to monitor babies receiving CPAP that they could not perform.^31^

A lack of comprehensive skills and the absence of a strategy to sustain the technology-related skills of health professionals had a detrimental impact on decisions regarding the use of CPAP in the Kenyan hospital study.^31^ Similarly, in the Malawian study on phototherapy, inadequate skills contributed to the insufficient use of phototherapy protocols to improve communication and inform decision-making. In the Malawian CPAP study, the lack of a systematic plan to disseminate training to all staff members resulted in inadequate skills among health professionals; the health professionals who had received CPAP training were reassigned to different departments before they had a chance to pass on their expertise, leaving behind replacements who lacked proper orientation in CPAP use.^31^ A study aimed at understanding the implementation of CPAP in four hospitals in Malawi found that the lack of clear roles and responsibilities among different groups of health professionals sometimes complicated communication processes. Despite nurses often receiving training, clinicians held greater authority in decision-making.^23^ This challenge of disagreement among health professionals regarding CPAP use was also reported in a paper that conducted a secondary analysis of data from the study originally conducted by Nyondo-Mipando *et al*.^24^

Inadequate financial support for essential equipment and supplies has been reported to influence decisions regarding technology use in two of the 14 studies. In the Malawian phototherapy study, a lack of transcutaneous bilirubinometers, a non‐invasive point-of-care device that estimates serum bilirubin levels, was found to influence health professionals to make decisions to use phototherapy without being aware of serum bilirubin levels.^35^ In the Indian study on the feasibility and acceptability of the paediatric pulse oximeter in six primary healthcare facilities, the authors found that none of the six primary healthcare centres had a paediatric pulse oximeter. Consequently, adult equipment was used for paediatric patients, posing challenges in obtaining and communicating accurate readings from the technology for monitoring babies.^29^ Lastly, interviews with 27 health professionals using continuous monitoring technologies in a newborn unit at a tertiary hospital in Kenya highlighted concerns, including a lack of technology replacement parts and insufficiently trained biomedical engineers for servicing and repairs. These issues discouraged the integration of the monitoring technology into their daily discussions and decision-making.^26^

### Technology characteristics

The review data suggest that health professionals’ communication associated with technologies depended on their perceptions of those technologies. Health professionals in neonatal units in Kenya believed that CPAP improved neonatal care when used appropriately. Positive personal experiences with CPAP boosted staff morale and confidence in communicating with colleagues and their decision to use it.^31^ Studies in India and the UK^28 30^ reported that perceptions of the difficulty of using a particular technology played a role in communication about its use among health professionals. In the Indian study, health professionals perceive CPAP as a less complicated technology than a ventilator, and, due to the perceived technical simplicity of using CPAP, most nurses and some doctors felt trained nurses could independently decide to initiate CPAP without discussions with doctors for approval.^28^ In the UK, paediatricians, midwives and neonatal nurses using a non-invasive neonatal heart rate monitoring device in three NICUs and one labour ward expressed a preference for a device that is ‘easy to use’, ‘easy to attach’, ‘self-explanatory’ and ‘easy to maintain’ for them to communicate and decide to use technology.^30^

## Discussion

Despite the crucial role of communication among health professionals who use newborn technologies to support care, our review found that few studies have explicitly addressed this topic. Many studies only included a few sentences on communication or focused on limited aspects associated with it. Consequently, there is a gap in achieving a comprehensive understanding of the role communication plays in technology implementation. Most studies were conducted in LMIC, with over half focusing on CPAP technology, leaving other technologies less explored.

This scant research evidence about communication relates to various health professionals, including nurses, doctors, respiratory therapists and healthcare managers involved in newborn technologies. However, other disciplines of health professionals playing a pivotal role in newborn care, such as dieticians^36 37^ and occupational therapists,^38 39^ were not reported in the evidence on technology-associated communication among health professionals in newborn care. We also indicate that the extent of the involvement of health professionals in communication varies based on their availability and autonomy. However, the literature on the timing and spatial aspects of these interactions is limited.

Greater reliance on technology has amplified the demand for an adequate number of health professionals. While studies on general communication among health professionals report on issues typically involving interactions among health professionals, including the challenge of inadequate staffing levels,^40^,^42^ this review reveals that technology use requires extra work, including documentation and procedural tasks. This additional work can affect communication among health professionals in the newborn unit.

This review also highlights the challenging infrastructure in LMICs, such as unreliable electricity, which led to negative decisions not to use newborn technologies. This challenge also led to increased communication between health professionals in newborn units and those outside the newborn unit who facilitated the availability of alternative energy, such as a generator. Further understanding of the physical environment of the newborn unit and its role in health professionals’ communication around technology use is needed. For example, evidence from the office setting shows that architectural design, such as workspace size, layout of windows, walls and cubicles and spatial proximity of coworkers, affects their interactions in an office setting.^43^ Hospitals with fewer wards, beds and buildings shortened distances between health professionals, improving communication and information flow.^44 45^ Understanding how the layout and physical structure affect communication among health professionals using technology in newborn care is crucial.

In the socio-organisational context, communication associated with technology use necessitates an additional layer of training and skills. This dual requirement underscores the complexity and additional burden placed on health professionals. Other factors, such as hierarchy, also influence communication, which is consistent with findings from general communication studies.^46 47^ While the studies in the scoping review did not provide an in-depth explanation, they provided an initial understanding of the culture and power dynamics and how they shape communication associated with technology. It also highlights a notable gap in the literature regarding a comprehensive exploration of other socio-organisational factors on communication in technology use, suggesting a need for further research.

In the scoping review, we report on how technology attributes, such as the design and how they function, can shape communication among health professionals. While the perceived difficulty of using technology deters health professionals from deciding to use it or fully participate in communication around its use, positive patient outcomes from it encourage them. However, there is limited evidence on how technology-specific factors influence communication among health professionals. Gaining deeper insights into technology attributes and their impact on communication among health professionals would be valuable.

### Strengths and limitations of the scoping review

The review strategy provided a comprehensive search of articles discussing communication in the newborn technology use context regardless of their quality. The limitation of the study is that it included only studies written in English, which excluded articles in other languages. Although the search strategies included varied terminologies, there was a possibility of missing technological studies that mentioned technical or proprietary names of technologies.

To address gaps in the literature, our team employs an ethnographic approach to examine how contextual factors shape communication among health professionals and, consequently, influence the use of newborn technologies. The findings will be published separately.

## Conclusions

Through this scoping review, we provide an overview of communication among health professionals using newborn technologies for care. We present notable gaps in the existing literature on communication in some groups of health professionals, such as dieticians, and a lack of in-depth understanding of interprofessional communication dynamics. The literature highlights the significance of physical and socio-organisational contexts on communication among health professionals. However, there remains a gap in information on additional contextual factors, such as the structure and layout of newborn facilities, and a comprehensive understanding of the impact of organisational culture and support on communication among health professionals. Given the limited empirical research on how technology attributes shape communication among health professionals, examination of structural and functional aspects of technology and comparative analyses within similar contexts to distinguish technology-specific issues from broader systemic factors is crucial.

## Supplementary material

10.1136/bmjoq-2025-003501 online supplemental file 1

## Data Availability

Data are available on reasonable request.
