# Supplementary material for: Communication among health professionals using newborn technology for care: an exploratory scoping review
Source: BMJ Open Qual. 2025 Sep 16;14(3):e003501. doi: 10.1136/bmjoq-2025-003501 (PMC12443173; doi:10.1136/bmjoq-2025-003501)
Supplement: online supplemental file 1 [file bmjoq-14-3-s001.docx]

**SUPPLEMENTARY DOCUMENTS**

**SUPPLEMENTARY MATERIAL 1: SCOPING REVIEW PROTOCOL**

**BACKGROUND**

Affordable newborn technologies such as a phototherapy machine and continuous positive airway pressure (CPAP) can significantly improve the quality of care and reduce preventable newborn deaths (1, 2). However, technologies can only work well and meet the intended aims with appropriate integration considering the health system’s complexity (3), context implementation dynamics (4), and social and organisational processes (5). Within this complexity, effective communication surrounding technology use is central to good clinical practice, enhanced patient outcomes, and improved interaction between families and health professionals (6).

Effective communication and participation are among the World Health Organisation’s (WHO) standards for improving the quality of care for small and sick newborn babies (7). Although the WHO standards emphasise the interaction between health professionals and families, effective communication among health professionals is essential. It nurtures competence and provides trust, kindness and good relationships among professionals and families (8).

Current evidence and efforts stress digital technologies, mainly information and communication technology (9-13). Little is known about communication associated with newborn technologies supporting other care aspects. The emphasis has also been on communication between health professionals and patients (14). There is scarce evidence of the interactions among various health professionals who engage with these technologies, particularly in LMIC newborn care.

This review explores the nature and extent of the literature on health professionals’ communication associated with low-cost newborn technology use, which supports care aspects other than information sharing.

**METHODS**

**Search Strategy**

The Joanna Briggs Institute Manual for Evidence Synthesis (15) and Preferred Reporting Items for Systematic review and Meta-Analysis extension for Scoping Reviews (PRISMA-ScR)(16) (attached below the protocol) informs the data collection and synthesis process.

The review answers these questions:

- What characterises communication among health professionals using newborn technologies in the hospital setting?
- What are the enablers and barriers to communication among health professionals using technologies in newborn care in the hospital setting?

We identified five keywords related to the review: communication, technology, health professionals, neonates, and health facilities.

We use the World Health Organisation’s definition of communication, which refers to providing information, guidance, and advice to take action to protect individuals’ health (17). Technology in this research is an innovation, a device, or a process introduced to change the existing practice. We also drew on the standardised definition of newborn medical devices (18) and innovations for improving newborn care (19). All health professionals working in neonatal care are included in this review. A neonate is a baby from the day of delivery up to 28 days of life (20). We considered health facilities at different levels, including primary health care centres, district/first referral, and specialised and general hospitals as defined by the World Health Organisation (21).

We will conduct the first round of relevant literature reviews between June and August 2021 and update the list if required. We will search seven databases: PubMed, Web of Science, Scopus, Embase, ERIC, Cochrane Library, and Google Scholar.

Table 1 below summarises information on the search terms:

| **Search term 1** | communication OR interaction* AND |
| --- | --- |
| **Search term 2** | “Health worker*” OR workforce* OR “human resource for health” OR “Healthcare worker*” OR nurse* OR health professional*AND |
| **Search term 3** | Technolog* OR innovation* AND |
| **Search term 4** | Neonat* OR “new?born*” OR “bab?*” OR “below 28 days” OR “less than 28 days” |
| **Search term 5** | Hospital* OR Ward* OR “Health cent?*” OR “Health OR “neonatal unit” OR “new?born unit” OR facilit*” |

Search terms used to identify articles on PubMed:

**(((("communication" OR "interaction*") AND ("Health professional*" OR "Health worker*" OR "workforce*" OR "human resource for health" OR "Healthcare worker*" OR "nurse*")) AND ("Technolog*" OR "innovation*" OR "medical?device*")) AND ("Neonat*" OR "new?born*" OR "bab?*" OR "below 28 days" OR "less than 28 days")) AND ("Hospital*" OR "ward*" OR "health cent?*" OR "neonatal unit" OR "new?born unit" OR facilit*")**

**Eligibility Criteria**

We have highlighted in Table 1 below the criteria for including studies in the review:

| **Articles Selection Criteria** | |
| --- | --- |
| Period | We will not put a time limit on the research data collection or article publication. |
| Document type | Grey literature, peer-reviewed and published literature.  Articles that discuss the use of low-cost technology in newborn care regardless of the geographical location. |
| Language | We will include only articles published in English. |
| Research method | Studies that used qualitative, quantitative, and mixed methods. |
| Quality | Since the review aim was to scope the available evidence, we will not consider the quality of the articles. |

Table 1: Scoping Review Criteria

**Data Charting and Synthesis**

We will develop a data collection tool on Microsoft Excel, which captures the studies’ characteristics, including the author (s), year of publication, geographical location, methodology, technology type, and limitations. Informed by previous studies, we will document findings based on identified essential areas in healthcare communication: groups of health professionals involved in the communication (22), communicating ways/modalities (22-24), information content (23, 25), time the communication took place and the quality (24) and factors affecting communication (22-24).

We will start the review by screening the article titles, followed by the abstract, to determine the articles' eligibility and remove duplicates. We will read full texts of articles that meet the inclusion criteria. We will read references of key articles to scan for other eligible articles. Since the review focuses on scoping the available literature on health professionals’ communication, we will include all articles regardless of the strength of the evidence.

We will extract data from all studies. Throughout the review process, GN (the first reviewer) will present data to the second, third, and fourth authors for quality assurance. Drawing on Braun and Clarke (2006)(26), we will familiarise ourselves with data, generate codes and themes, and review and define themes to represent findings.

**REFERENCES**

1. Rosa-Mangeret F, Benski A-C, Golaz A, Zala PZ, Kyokan M, Wagner N, et al. 2.5 Million Annual Deaths-Are Neonates in Low- and Middle-Income Countries Too Small to Be Seen? A Bottom-Up Overview on Neonatal Morbi-Mortality. Tropical medicine and infectious disease. 2022;7(5):64.

2. Bhutta ZAP, Das JKMBA, Bahl RP, Lawn JEP, Salam RAM, Paul VKMD, et al. Can available interventions end preventable deaths in mothers, newborn babies, and stillbirths, and at what cost? Lancet. 2014;384(9940):347-70.

3. Greenhalgh T, Papoutsi C. Studying complexity in health services research: desperately seeking an overdue paradigm shift. BMC Medicine. 2018;16(1):95.

4. May CR, Johnson M, Finch T. Implementation, context and complexity. Implement Sci. 2016;11(1):141-.

5. Blaauw D, Gilson L, Penn-Kekana L, Schneider H. Organisational Relationships and the ‘Software'of Health Sector Reform. Centre for health policy. 2003.

6. O'Daniel M, Rosenstein A. Professional Communication and team collaboration patient safety and quality: An eëidence-Based handbook for nurses. Agency for Healthcare Research and Quality (US). 2008:271-84.

7. World Health Organisation. Standards for improving the quality of care for small and sick newborns in health facilities. Geneva: World Health Organization; 2020.

8. Busari JO, Moll FM, Duits AJ. Understanding the impact of interprofessional collaboration on the quality of care: A case report from a small-scale resource limited health care environment. J Multidiscip Healthc. 2017;10:227-34.

9. Duran P, Sommer JA, Otero P, Daus M, Benitez S, Serruya S, et al. Information and communication technologies in neonatal health. 2020;(1680-5348 (Electronic)).

10. Castiglia P, Dettori M, Arghittu A, Campus G, De Vito D. Use of digital technologies in public health: a narrative review. Acta Biomedica Atenei Parmensis. 2023;94(S3):e2023164.

11. Martin G, Khajuria A, Arora S, King D, Ashrafian H, Darzi A. The impact of mobile technology on teamwork and communication in hospitals: a systematic review. 2019.

12. World Health Organisation. Global Strategy on Digital Health 2020-2025. <https://wwwwhoint/docs/default-source/documents/gs4dhdaa2a9f352b0445bafbc79ca799dce4dpdf> 2021. Accessed online on 23/110/2023.

13. Duran P, Sommer JA, Otero P, Daus M, Benitez S, Serruya S, et al. Information and communication technologies in neonatal health. Rev Panam Salud Publica. 2020;44(123):e123-e.

14. Tenfelde K, Antheunis M, Krahmer E, Bunt JE. Using Digital Communication Technology to Improve Neonatal Care: Two-Part Explorative Needs Assessment. JMIR Pediatr Parent. 2023;6:e38435-e.

15. Peters MDJ, Marnie C, Tricco AC, Pollock D, Munn Z, Alexander L, et al. Updated methodological guidance for the conduct of scoping reviews. JBI Evidence Synthesis. 2020;18(10).

16. Tricco AC, Lillie E, Zarin W, O'Brien KK, Colquhoun H, Levac D, et al. PRISMA Extension for Scoping Reviews (PRISMA-ScR): Checklist and Explanation. Annals of internal medicine. 2018;169(7):467-73.

17. World Health Organisation. World Health Organisation Strategic Communications Framework https://wwwwhoint/docs/default-source/documents/communicating-for-health/communication-frameworkpdf. 2017. [Accessed online on 22/11/2023]

18. Lawn JE, Ohuma EO, Bradley E, Idueta LS, Hazel E, Okwaraji YB, et al. Small babies, big risks: global estimates of prevalence and mortality for vulnerable newborns to accelerate change and improve counting. 2023.

19. Lunze K, Higgins-Steele A, Simen-Kapeu A, Vesel L, Kim J, Dickson K. Innovative approaches for improving maternal and newborn health - A landscape analysis. BMC Pregnancy and Childbirth. 2015;15(1):337.

20. World Health Organisation. Newborn Mortality: Key Facts. <https://wwwwhoint/news-room/fact-sheets/detail/levels-and-trends-in-child-mortality-report-2021#:~:text=Sub%2DSaharan%20Africa%20has%20the,36%25%20of%20global%20newborn%20deaths>. 2022. Accessed online on 13/09/2023. 2022.

21. World Health Organisation. Guidelines on Hand Hygiene in Health Care: First Global Patient Safety Challenge Clean Care Is Safer Care. Geneva: World Health Organization; 2009. Appendix 1, Definitions of health-care settings and other related terms. Available from: <https://wwwncbinlmnihgov/books/NBK144006/>. 2009. [Accessed online on 07/11/2023].

22. Oliveros E, Brailovsky Y, Shah KS. Communication Skills: The Art of Hearing What Is Not Said. JACC Case reports. 2019;1(3):446-9.

23. Vermeir P, Vandijck D, Degroote S, Peleman R, Verhaeghe R, Mortier E, et al. Communication in healthcare: a narrative review of the literature and practical recommendations. 2015.

24. Chichirez CM, Purcărea VL. Interpersonal communication in healthcare. Journal of medicine and life. 2018;11(2):119-22.

25. Schroeder RE, Morrison Ee Fau - Cavanaugh C, Cavanaugh C Fau - West MP, West Mp Fau - Montgomery J, Montgomery J. Improving communication among health professionals through education: a pilot study. 1999(0735-6722 (Print)).

26. Braun V, Clarke V. Using thematic analysis in psychology. Qualitative research in psychology. 2006;3(2):77-101.

**SUPPLEMENTARY MATERIAL 2: PREFERRED REPORTING ITEMS FOR SYSTEMATIC REVIEWS AND META-ANALYSES EXTENSION FOR SCOPING REVIEWS (PRISMA-ScR) CHECKLIST**

| **SECTION** | **ITEM** | **PRISMA-ScR CHECKLIST ITEM** | **REPORTED ON PAGE #** |
| --- | --- | --- | --- |
| **TITLE** | | | |
| Title | 1 | Identify the report as a scoping review. | 1 |
| **ABSTRACT** | | | |
| Structured summary | 2 | Provide a structured summary that includes (as applicable): background, objectives, eligibility criteria, sources of evidence, charting methods, results, and conclusions that relate to the review questions and objectives. | 1, 2 |
| **INTRODUCTION** | | | |
| Rationale | 3 | Describe the rationale for the review in the context of what is already known. Explain why the review questions/objectives lend themselves to a scoping review approach. | 3 |
| Objectives | 4 | Provide an explicit statement of the questions and objectives being addressed with reference to their key elements (e.g., population or participants, concepts, and context) or other relevant key elements used to conceptualize the review questions and/or objectives. | 3 |
| **METHODS** | | | |
| Protocol and registration | 5 | Indicate whether a review protocol exists; state if and where it can be accessed (e.g., a Web address); and if available, provide registration information, including the registration number. | 3 |
| Eligibility criteria | 6 | Specify characteristics of the sources of evidence used as eligibility criteria (e.g., years considered, language, and publication status), and provide a rationale. | 4 |
| Information sources* | 7 | Describe all information sources in the search (e.g., databases with dates of coverage and contact with authors to identify additional sources), as well as the date the most recent search was executed. | 4 |
| Search | 8 | Present the full electronic search strategy for at least 1 database, including any limits used, such that it could be repeated. | 4 |
| Selection of sources of evidence | 9 | State the process for selecting sources of evidence (i.e., screening and eligibility) included in the scoping review. | 4 |
| Data charting process | 10 | Describe the methods of charting data from the included sources of evidence (e.g., calibrated forms or forms that have been tested by the team before their use, and whether data charting was done independently or in duplicate) and any processes for obtaining and confirming data from investigators. | 5 |
| Data items | 11 | List and define all variables for which data were sought and any assumptions and simplifications made. | 5 |
| Critical appraisal of individual sources of evidence | 12 | If done, provide a rationale for conducting a critical appraisal of included sources of evidence; describe the methods used and how this information was used in any data synthesis (if appropriate). | N/A |
| Synthesis of results | 13 | Describe the methods of handling and summarizing the data that were charted. | 5 |
| **RESULTS** | | | |
| Selection of sources of evidence | 14 | Give numbers of sources of evidence screened, assessed for eligibility, and included in the review, with reasons for exclusions at each stage, ideally using a flow diagram. | 4 |
| Characteristics of sources of evidence | 15 | For each source of evidence, present characteristics for which data were charted and provide the citations. | 7,8 |
| Critical appraisal within sources of evidence | 16 | If done, present data on critical appraisal of included sources of evidence (see item 12). | N/A |
| Results of individual sources of evidence | 17 | For each included source of evidence, present the relevant data that were charted that relate to the review questions and objectives. | 9 - 15 |
| Synthesis of results | 18 | Summarize and/or present the charting results as they relate to the review questions and objectives. | 6, 9 - 15 |
| **DISCUSSION** | | | |
| Summary of evidence | 19 | Summarize the main results (including an overview of concepts, themes, and types of evidence available), link to the review questions and objectives, and consider the relevance to key groups. | 15-16 |
| Limitations | 20 | Discuss the limitations of the scoping review process. | 17 |
| Conclusions | 21 | Provide a general interpretation of the results with respect to the review questions and objectives, as well as potential implications and/or next steps. | 17 |
| **FUNDING** | | | |
| Funding | 22 | Describe sources of funding for the included sources of evidence, as well as sources of funding for the scoping review. Describe the role of the funders of the scoping review. | 17 |

JBI = Joanna Briggs Institute; PRISMA-ScR = Preferred Reporting Items for Systematic reviews and Meta-Analyses extension for Scoping Reviews.

* Where *sources of evidence* (see second footnote) are compiled from, such as bibliographic databases, social media platforms, and Web sites.

† A more inclusive/heterogeneous term used to account for the different types of evidence or data sources (e.g., quantitative and/or qualitative research, expert opinion, and policy documents) that may be eligible in a scoping review as opposed to only studies. This is not to be confused with *information sources* (see first footnote).

‡ The frameworks by Arksey and O’Malley (6) and Levac and colleagues (7) and the JBI guidance (4, 5) refer to the process of data extraction in a scoping review as data charting*.*

§ The process of systematically examining research evidence to assess its validity, results, and relevance before using it to inform a decision. This term is used for items 12 and 19 instead of "risk of bias" (which is more applicable to systematic reviews of interventions) to include and acknowledge the various sources of evidence that may be used in a scoping review (e.g., quantitative and/or qualitative research, expert opinion, and policy document).

*From:* Tricco AC, Lillie E, Zarin W, O'Brien KK, Colquhoun H, Levac D, et al. PRISMA Extension for Scoping Reviews (PRISMAScR): Checklist and Explanation. Ann Intern Med. 2018;169:467–473. [doi: 10.7326/M18-0850](http://annals.org/aim/fullarticle/2700389/prisma-extension-scoping-reviews-prisma-scr-checklist-explanation).
